# Platelet-Derived Extracellular Vesicles for the Diagnosis of Ovarian Cancer

**DOI:** 10.3390/cancers18213478

**Published:** 2026-10-28

**Authors:** Evelina Karlsson, Katja Gabrysch, Karin Stålberg, Inger Sundström-Poromaa, Fariborz Mobarrez, Marta Lomnytska

**Affiliations:** 1 Department of Women’s and Children’s Health, Uppsala University, SE-751 85 Uppsala, Sweden; evelina.karlsson@uu.se (E.K.); karin.stalberg@uu.se (K.S.); inger.sundstrom@uu.se (I.S.-P.); 2 Uppsala Clinical Research Center, Uppsala University, SE-751 85 Uppsala, Sweden; katja.gabrysch@ucr.uu.se; 3 Department of Medical Sciences—Clinical Chemistry, Uppsala University, SE-751 85 Uppsala, Sweden; fariborz.mobarrez@uu.se

**Keywords:** ovarian cancer, platelet-derived extracellular vesicles, extracellular vesicles, platelet biomarkers, gelsolin, flow cytometry, diagnostic biomarkers

## Abstract

**Objective**: Differentiating ovarian cancer from benign and borderline adnexal lesions remains challenging. We previously identified nine platelet protein markers associated with ovarian cancer. This study aimed to validate these markers on platelet-derived extracellular vesicles (EVs) and assess their ability to distinguish ovarian cancer from benign and borderline adnexal lesions. **Methods**: Patients were recruited between 2021 and 2025 at Uppsala University Hospital and Gävle Hospital, Sweden, and grouped according to postoperative histopathological diagnosis. Platelet-free plasma samples were analyzed by flow cytometry. Platelet-derived EVs (CD41+) and nine platelet protein markers (ACTN4, CRKL, ERP29, GELS, MAP1A, MYCT1, PHB, RELN, and SRC) were quantified in CD41+CD62P+ and CD41+CD62P− EV populations. Group differences were assessed using non-parametric tests, binary and proportional-odds logistic regression, and Boruta feature selection. **Results**: The total CD41+ EVs and CD41+CD62P+ EVs did not differ between groups. The levels of CD62P+anti-GELS, CD62P+anti-SRC, and CD62P−anti-MYCT1 were highest in ovarian cancer. CD62P+anti-GELS was independently associated with ovarian cancer in both crude analysis (OR: 1.47, 95% CI: 1.16–1.85; C-statistic 0.63) and analysis adjusted for age and CA125 (OR: 1.59, 95% CI: 1.21–2.08; C-statistic 0.82). The Boruta algorithm identified ultrasound assessment and CA125, followed by CD62P+anti-GELS, CD62P−anti-SRC, CD62P−anti-ERP29, CD62P−anti-MAP1A, and CD62P+anti-ACTN4, as the variables associated with ovarian cancer. CD62P+anti-ERP29 was specifically associated with advanced-stage ovarian cancer compared with other abdominal malignancies (OR: 2.43, 95% CI: 1.53–4.38; C-statistic 0.85). **Conclusion**: CD62P+anti-GELS on platelet EVs was independently associated with ovarian cancer after adjustment for age and CA125. Together with CD62P+/CD62P− anti-SRC, CD62P+anti-ACTN4, CD62P−anti-ERP29 and CD62P−anti-MAP1A, it may complement ultrasound evaluation and CA125 in distinguishing ovarian cancer from benign and borderline adnexal lesions. The findings require further validation.

## Simple Summary

The differential diagnosis of ovarian cancer from benign and borderline adnexal lesions remains challenging. This study evaluated whether platelet protein markers on platelet-derived extracellular vesicles (EVs) can distinguish ovarian cancer from benign and borderline adnexal lesions. Blood samples from patients at Uppsala University Hospital and Gävle Hospital (2021–2025) were analyzed for nine platelet protein markers that we had previously identified. While overall, CD41+ and CD41+CD62P+ EV levels did not differ between patient groups, the markers CD62P+anti-GELS, CD62P+anti-SRC and CD62P−anti-MYCT1 showed higher levels in ovarian cancer. In regression analyses, CD62P+anti-GELS was the only marker independently associated with ovarian cancer, even after adjusting for age and CA125. The Boruta algorithm identified ultrasound assessment and CA125, followed by CD62P+anti-GELS, CD62P−anti-SRC, CD62P−anti-ERP29, CD62P−anti-MAP1A, and CD62P+anti-ACTN4, as the variables associated with ovarian cancer. In regression analyses, CD62P+anti-ERP29 was exclusively associated with advanced-stage ovarian cancer compared with other abdominal malignancies. These findings suggest that platelet EV protein markers, particularly CD62P+anti-GELS, are promising candidates that may complement ultrasound assessment and CA125 testing to improve ovarian cancer diagnosis after validation.

## 1. Introduction

Despite advances in diagnostics and treatment, ovarian cancer is the second-highest cause of death among all gynecological cancers worldwide. Approximately 75% of ovarian cancers are diagnosed at advanced stages, with the estimated number of new cases in Europe in 2020 being 66 693, with 44 053 deaths [1]. Among patients who present with adnexal lesions, transvaginal ultrasound using specific image criteria provides the highest sensitivity and specificity for diagnosis of adnexal lesions and ovarian cancer [2,3,4]. However, in up to 20% of cases, the evaluation remains inconclusive [5]. Over the years, several attempts to develop screening methods for ovarian cancer have been undertaken, yet none has survived translation into clinical practice [6].

Platelets represent an attractive source of biomarker candidates. Platelets have been demonstrated to be important for cancer metastases in several ways: platelets protect cancer cells from the immune system by coating them, thus helping them hide from natural killer cells [7,8,9,10,11,12]; they mediate signaling and cancer spread [13,14]; and they promote epithelial–mesenchymal transition [15]. In addition, platelets transport growth factors to the endothelium and to cancer cells, thus promoting angiogenesis and metastatic spread [13]. Of particular relevance to the present study, platelet activation has been demonstrated in patients with ovarian cancer [16]. The activation is thought to occur either by direct binding to the platelets or, indirectly, by secretion of cytokines and proteins that trigger platelet activation [17].

Using proteomics, we have identified platelet protein markers capable of differentiating between benign ovarian lesions and cancer, with 96% sensitivity and 88% specificity, and predicting ovarian cancer stages I–II [18]. These platelet biomarkers were also detected on platelet extracellular vesicles (EVs) and were able to differentiate benign adnexal lesions from borderline tumors and ovarian cancer of stages I–II and III–IV with AUC > 0.9 [19]. Subsequent platelet proteome studies, using quantitative mass spectrometry, have identified additional markers with 93% detection accuracy for ovarian cancer compared with benign cases, and the proteins MYCT1, MAP1A and RELN were also subsequently detected on the platelet EVs (manuscript in submission). EVs can be analyzed in frozen platelet-free plasma without platelet isolation and are pre-analytically more robust, and CD62P status allows assessment of platelet activation in the same measurement [20]. Various cells during their activation or apoptosis release EVs carrying part of the cell membrane and bioactive molecules into the bloodstream [21]. Around 60% of EVs originate from platelets [22]. Platelet-derived EVs are largely formed by budding from the activated platelet membrane and are well represented in the large EV fraction. Platelet-derived EVs can themselves promote metastasis [23] and are important for communication between cells [24].

The aim of this study was to evaluate nine of our earlier identified platelet protein markers of ovarian cancer on platelet EVs for their diagnostic potential in ovarian cancer.

## 2. Materials and Methods

### 2.1. Study Population

In this cross-sectional study, study participants were recruited between December 2021 and May 2025 during their visit before surgery at Uppsala University Hospital and Gävle Regional Hospital, Sweden. The inclusion criteria were planned surgery for presumed benign, borderline adnexal lesions and primary epithelial ovarian cancer of all FIGO stages and at least 18 years of age. Postoperative histopathology was mandatory for stratification of patients into study groups (Table 1). The exclusion criteria were a known active other malignancy (except preinvasive cervical lesions and radically excised squamous-cell cancer of the skin), surgical histology not being available, and psychiatric disease or substance abuse possibly interfering with compliance to participate. Patients whose postoperative histology revealed malignancies of other origins were included as a comparison group with the ovarian cancer stages III–IV. All participants received written information and time to discuss this study and provided written informed consent. This study was approved by the Swedish Ethical Review Authority (Dnr 2020-06596 from 28 January 2021 and Dnr 2022-07236-02 from 23 January 2023).

### 2.2. Study Variables

The baseline variables were age, cancer antigen 125 (CA125) and ultrasound features, both subjectively classified [2] according to the International Ovarian Tumour Analysis (IOTA) group’s simple rules, classifying lesions into benign, inconclusive and malignant, based on five features typical of benign tumors (B-features) and five features typical of malignant tumors (M-features) [3,25]. The outcome data were histopathology and FIGO stages for ovarian cancer.

### 2.3. Sample Collection and Preparation

Venous blood was collected in citrate tubes and, within one hour of sample collection, centrifuged at 2000× *g* for 20 min at room temperature. Thereafter, plasma was collected in Eppendorf tubes and once more centrifuged at 2000× *g* for 20 min, resulting in platelet-free plasma (PFP). These tubes were then frozen and stored at −70 °C.

### 2.4. Flow Cytometry Analysis of Extracellular Vesicles

PFP samples were thawed in a 37 °C water bath for approximately five minutes, vortexed briefly and centrifuged at 2000× *g* for 20 min at room temperature. The supernatant was transferred to a new tube and centrifuged at 13,000× *g* for two minutes to remove remaining cells and debris. An amount of 20 µL of the resulting PFP was then added to each of three wells of a 96-well plate containing one of three antibody cocktails (Table S1). Each cocktail contained anti-CD41 as a platelet marker and anti-CD62P, or P-selectin, a marker of platelet activation, together with antibodies against three of the nine candidate proteins. We analyzed nine proteins. The proteins were alpha-actinin-4 (ACTN4), Crk-like protein (CRKL), endoplasmic reticulum resident protein 29 (ERP29), gelsolin (GELS), prohibitin (PHB), proto-oncogene tyrosine-protein kinase Src (SRC), MYC target protein 1 (MYCT1), microtubule-associated protein 1A (MAP1A) and reelin (RELN) (Table S1C). CD41 is constitutively expressed on platelets and megakaryocytes and their EVs, whereas CD62P is stored in α-granule membranes and exposed on the surface upon platelet activation; subdivision into CD62P+ and CD62P− populations, therefore, allowed assessment of whether the candidate proteins are associated with activation-dependent EV release. CD41 and CD62P antibodies were factory-conjugated (Beckman Coulter, Brea, CA, USA); the nine anti-protein antibodies (Abcam, Cambridge, UK) were conjugated in-house using Lightning-Link kits. The plate was incubated in the dark for 20 min at room temperature, after which 120 µL of CytoFLEX sheath fluid was added to each well.

EVs were analyzed using a CytoFLEX flow cytometer (Beckman Coulter) with acquisition triggered on violet side scatter. The EV gate was defined using Spherotech Nano Fluorescent Yellow particles of 0.22, 0.45, 0.88 and 1.35 µm (Spherotech, Lake Forest, IL, USA); events within the approximately 0.2-to-1.0 µm bead-equivalent range were considered EVs. This study focused on large EVs; small EVs (<200 nm) were not analyzed, and the findings refer to large platelet-derived EVs. Positivity gates for each fluorochrome were set using unstained samples and fluorescence-minus-one (FMO) controls, with single-stained samples used for compensation. The vesicular nature of the detected events was verified by detergent lysis (Triton X-100), with buffer-only and antibody-only controls used to exclude reagent-derived signals. Platelet-derived EVs were defined as CD41+ events and subdivided into CD62P+ and CD62P− populations, within which the nine protein markers were quantified. The results are expressed as the fraction of positive events within the EV gate for CD41, and as fractions of the CD41+CD62P+ and CD41+CD62P− populations for the nine markers. Samples were analyzed in three batches (2023, spring 2025 and summer 2025), each time bridging with samples across the batches.

### 2.5. Statistical Analysis

Comparison was performed between patient groups as follows: benign, borderline, ovarian cancer stages I–II and ovarian cancer stages III–IV, with separate subgroup analyses of endometriosis and other abdominal malignancies. An unordered comparison between patient groups was performed using the Kruskal–Wallis, Mann–Whitney test and Pearson’s χ2 test. Assuming the following order of the patient groups as benign < borderline < ovarian cancer stage I–II < ovarian cancer stage III–IV, the predictive value of platelet EV biomarkers across patient groups was evaluated using a proportional odds logistic regression, providing odds ratios and 95% confidence intervals.

Furthermore, Boruta algorithms were applied to rank variables by importance associated with ovarian cancer detection. In Boruta, variables that performed better or worse than random noise were classified as confirmed or rejected. Hence, if a biomarker is confirmed, it means that the biomarker contributed to the model classifying the patient correctly. If not confirmed or rejected, the Boruta algorithm is indecisive, and variables are classified as tentative. This approach captures all relevant variables instead of only non-redundant variables; thus, correlated variables may be included. Furthermore, the Boruta algorithm identifies both linear and non-linear associations between a biomarker and an outcome [26,27]. Ordinal logistic regression and the Boruta algorithm were performed in unadjusted and adjusted models including CA125, age, and either subjective ultrasound or IOTA simple rules. In adjusted models, missing values of covariates were handled by multiple imputations using the chained equations (MICE) approach with the predictive mean matching (PMM) method. We generated 20 imputed datasets, and the estimates in proportional-odds logistic regression were calculated by Rubin’s rule. All biomarkers were log2-transformed before analysis. Binary logistic regression was used for comparison between cases with endometriosis and other benign adnexal lesions and between ovarian cancer stage III–IV and other abdominal malignancies. A *p*-value of <0.05 was considered significant. To avoid type I errors, Bonferroni correction was applied. For the statistical analyses, RStudio 4.4.1. was used.

The Microsoft 365 Copilot (GPT-5-powered AI assistant, Microsoft Corporation, Redmond, WA, USA) was used for the purposes of the grammar check and creation of the graphical abstract. The content was carefully revised. 

## 3. Results

### 3.1. Patient Characteristics

This study included 154 patients. Of 63 patients with benign lesions, 16 (25.4%) had endometriosis. Additionally, 19 patients had a different abdominal malignancy (pseudomyxoma peritonei (n = 3), leiomyosarcoma (n = 1), uterine stroma-cell sarcoma (n = 1), endometrial cancer (n = 2), gastrointestinal and colon cancer (n = 8), granulosa-cell ovarian cancer (n = 2) and appendix cancer (n = 2)). The baseline characteristics of the patients with benign or borderline lesions and ovarian cancer are presented in Table 1.

Age varied among patient groups, being lowest in patients with benign lesions and stage I–II ovarian cancer (*p* = 0.019). CA125 gradually increased from the lowest value in patients with benign adnexal lesions to the highest in ovarian cancer stage III–IV (*p* < 0.001) (Table 1).

### 3.2. Outcomes of Gynecologic Ultrasound

Lesions were classified by subjective ultrasound examination and IOTA as malignant in 78.1% and 76.5% of cases of ovarian cancer stage III–IV, respectively, but only in 38.5% and 46.2% of cases of ovarian cancer stage I–II. Ovarian cancer stage I–II was assessed as borderline in 38.5% of cases and as uncertain in 7.7% and 38.5% of cases by subjective ultrasound and IOTA, respectively. Benign adnexal lesions were assessed as benign by subjective ultrasound and IOTA in 65.0% and 60.7%, respectively (*p* < 0.001) (Table 1).

### 3.3. Levels of Platelet Protein Markers on the Platelet Extracellular Vesicles

An unordered comparison of patient groups demonstrated no difference in the levels of CD41+ EVs (*p* = 0.454) or CD41+CD62P+ EVs (*p* = 0.307) between patient groups (Table 2).

The differences in the levels of CD62P+anti-GELS (*p* = 0.005), CD62P+anti-SRC (*p* = 0.026), and CD62P−anti-MYCT1 (*p* = 0.009) and the marginal difference in CD62P− anti-ACTN4 (*p* = 0.050) between patient groups were not significant after the Bonferroni correction (Table 2).

### 3.4. Performance of Platelet Biomarkers Together with CA125 and Ultrasound Evaluation

After the initial unordered comparison of patient groups, we performed proportional-odds regression analysis based on the predefined disease progression order: benign < borderline < ovarian cancer stage I–II < ovarian cancer stage III–IV (Table 3).

Higher levels of CD62P+anti-GELS, CD62P+anti-ACTN4 and CD62P+anti-SRC were nominally associated with increased odds of belonging to a more advanced disease category. After Bonferroni correction, only CD62P+anti-GELS remained significantly associated with disease severity (OR = 1.47 [95% CI: 1.16–1.85]; C-statistics 0.63). After adjustment for age and CA125, the association remained significant (OR = 1.59 [95% CI: 1.21–2.08]; C-statistics 0.82) (Table 3). After adjustment for age, CA-125 and either subjective ultrasound or ultrasound by IOTA, the association for CD62P+anti-GELS was attenuated and did not remain significant after Bonferroni correction (Table S2).

**Figure 1:**
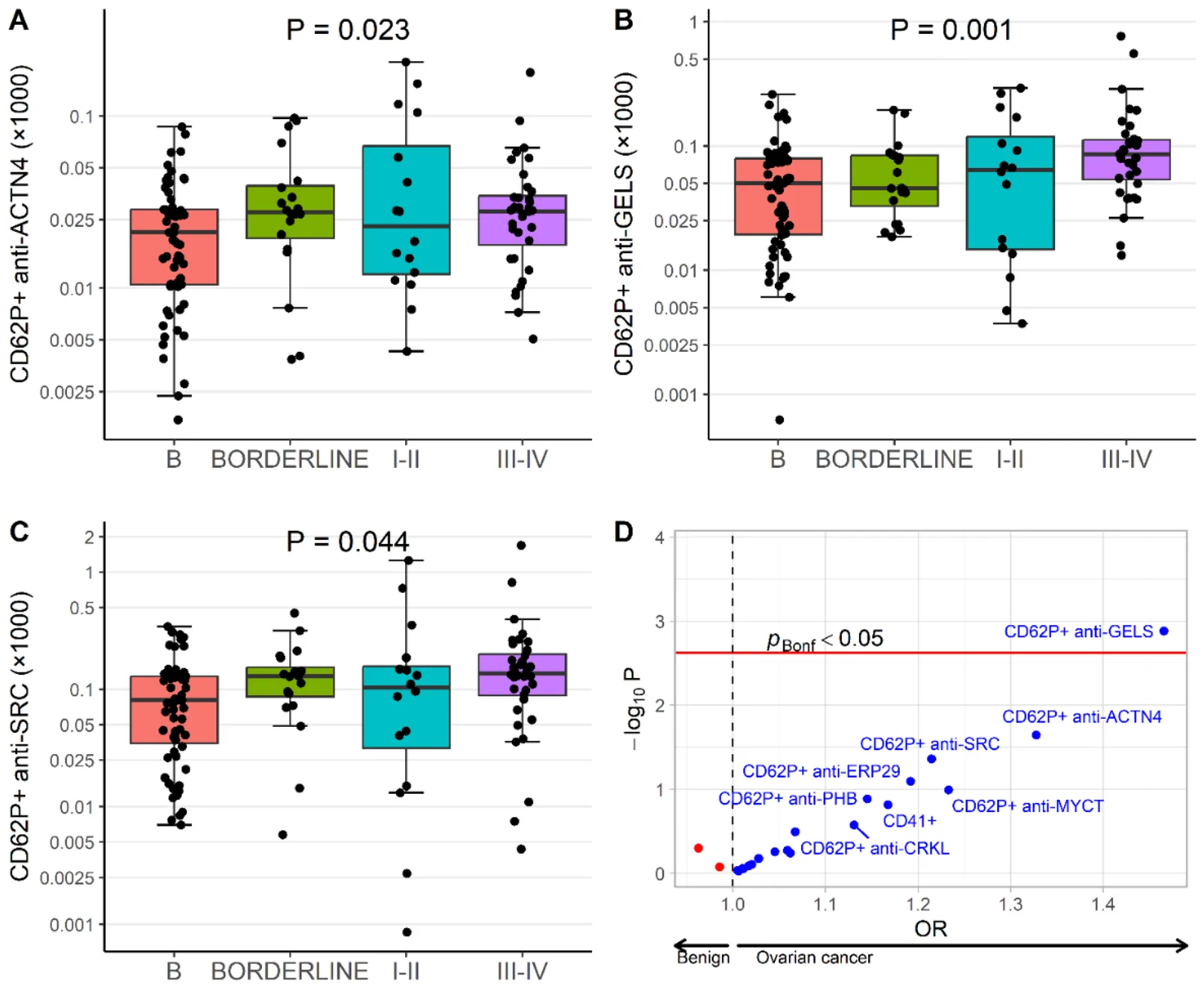
Expression of studied proteins for each patient group: (**A**) CD62P+ anti-ACTN4, (**B**) CD62P+ anti-GELS, and (**C**) CD62P+ anti-SRC. Raw values for each patient group; *p*-values originate from unadjusted ordinal logistic regression. (**D**) Volcano plot of odds ratios and *p*-values from ordinal regression model. Red line indicates a significant *p*-value after Bonferroni correction.

Median levels of CD62P+ anti-ACTN4, CD62P+ anti-GELS and CD62P+ anti-SRC in the patient groups are shown in Figure 1A–C (Table 3; Figure 1D).

**Figure 2:**
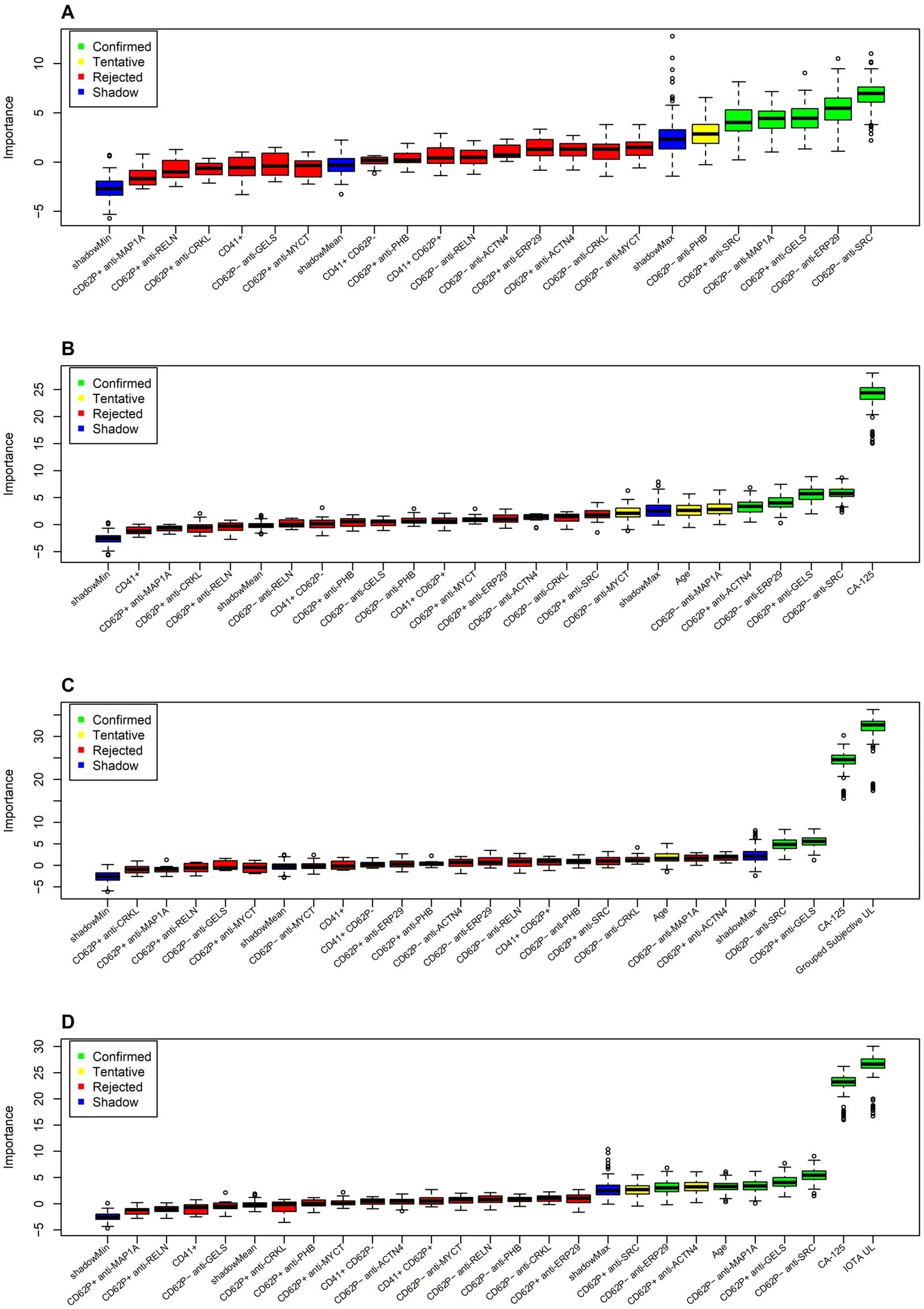
Classification of biomarkers in Boruta algorithm and their level of importance for discrimination between patient groups, including (**A**) platelet markers on EVs; (**B**) platelet markers on EVs, age and CA125; (**C**) platelet markers on EVs, age, CA125 and subjective ultrasound evaluation; and (**D**) platelet markers, age, CA125 and IOTA ultrasound.

To assess the contribution of platelet EV markers as part of a biomarker panel, we applied the Boruta feature selection algorithm. Among platelet-derived EV markers, CD62P+anti-GELS, CD62P+anti-SRC, CD62P−anti-SRC, CD62P−anti-ERP29 and CD62P−anti-MAP1A were identified as important variables for classification of the patient groups (Figure 2A). When CA125 was added to the model, it ranked highest, followed by CD62P+anti-GELS, CD62P+anti-ACTN4, CD62P−anti-SRC and CD62P− anti-ERP29 (Figure 2B). Adding subjective ultrasound and IOTA identified that the latter were superior to CA125, while other confirmed markers were CD62P+anti-GELS, CD62P−anti-SRC, CD62P− anti-MAP1A, age, and CD62P−anti-ERP29 (Figure 2C,D). In the Boruta algorithm restricted to benign lesions, borderline tumors and stage I–II ovarian cancer (Figure S1A), adjusted for CA125 (Figure S1B), CA125 and subjective ultrasound (Figure S1C) and CA-125 and IOTA classification (Figure S1D), CD62P−anti-SRC, CD62P+anti-ACTN4, CD62P−anti-ERP29 and CD62P−anti-MAP1A were confirmed, but not CD62P+anti-GELS.

### 3.5. Biomarkers on Platelet Extracellular Vesicles in Endometriosis and Other Cancers

Among patients with benign adnexal lesions, those with endometriosis showed higher levels of CD62P+anti-PHB than patients with other benign lesions (*p* = 0.047). However, this association did not remain significant after Bonferroni correction (Table 4A).

Logistic regression analysis similarly demonstrated an association between CD62P+anti-PHB and endometriosis (OR: 1.55, 95% CI: 1.08–2.52; C-statistic 0.67), which was no longer significant after correction for multiple testing (Table S3).

In contrast, markedly elevated levels of CD62P+anti-ERP29 were observed in patients with stage III–IV ovarian cancer compared with patients with other intra-abdominal malignancies (Bonferroni-corrected *p* < 0.001; Table 4B). This finding was confirmed in logistic regression analysis (OR: 2.43, 95% CI: 1.53–4.38; C-statistic: 0.85) and remained significant after correction for multiple testing (Table S4). These results indicate that CD62P+anti-ERP29 may be useful for distinguishing advanced ovarian cancer from other malignancies involving the abdominal cavity.

## 4. Discussion

Validation of our previously identified platelet protein markers demonstrated that CD62P+anti-GELS on platelet-derived EVs was independently associated with ovarian cancer, even after adjustment for age and CA125. Furthermore, CD62P+anti-GELS, together with CD62P+/CD62P− anti-SRC, CD62P+anti-ACTN4, CD62P−anti-ERP29, and CD62P−anti-MAP1A, emerged as candidate markers that may complement ultrasound evaluation and CA125 in distinguishing ovarian cancer from benign and borderline adnexal lesions.

In the present study, we evaluated a panel of platelet markers of ovarian cancer previously identified through platelet proteomic analyses, but here measured on platelet-derived EVs subdivided into CD62P+ and CD62P− fractions. Our findings suggest that platelet EV-associated proteins contain diagnostic information that may contribute to improved differentiation between benign, borderline, and malignant adnexal lesions.

Our results further support the growing evidence that platelets contribute to cancer progression [9,10,12,13], promotion of epithelial–mesenchymal transition [15], and enhancement of angiogenesis and metastasis spread [13]. The observed changes in platelet EV-associated proteins may, therefore, reflect biological interactions between tumor cells and circulating platelets during ovarian cancer development.

Among all evaluated markers, CD62P+anti-GELS emerged as the strongest candidate biomarker. We identified an increased expression of CD62P+anti-GELS from benign adnexal lesions and toward ovarian cancer, being the strongest candidate for ovarian cancer biomarkers. GELS, or gelsolin, is an actin-binding protein involved in cytoskeletal remodeling through regulation of actin filament assembly and disassembly, thus important for cytoskeleton remodeling due to its function in severing actin filaments [28]. The activity of the protein is regulated by several mechanisms; one of those is related to Ca^2+^ [29]. Previous studies have identified circulating plasma gelsolin as a potential biomarker for early ovarian cancer detection and prognosis [30]. Furthermore, exosome-associated gelsolin has been implicated in ovarian cancer chemoresistance [31,32]. Our findings extend these observations by demonstrating increased expression of gelsolin on activated platelet-derived EVs in ovarian cancer, supporting its potential diagnostic relevance. SRC was another promising marker identified in our analyses. SRC is a proto-oncogenic tyrosine kinase that has been reported to be overexpressed in advanced ovarian cancer and to play an important role in tumor progression [33,34,35]. Moreover, SRC protein has been demonstrated to be important for platelet activation [36]. SRC was identified in alpha granules [37], whereas we identified SRC on platelet-derived EVs, with increased levels in ovarian cancer. Although SRC expression did not remain significant after adjustment for multiple testing, both regression and Boruta analyses consistently highlighted SRC-associated EV markers, suggesting that further investigation is warranted.

Additional candidate markers included ACTN4, ERP29, and MAP1A.

ACTN4 is a cytoskeletal protein involved in actin organization and has previously been associated with metastatic potential through its involvement in cell motility, while its overexpression is associated with increased cell migration [38,39]. In ovarian cancer, ACTN4 has been demonstrated as over-expressed in advanced stages and associated with higher chemoresistance and a poorer outcome [40,41]. ACTN4 was earlier identified in EVs [37].

ERP29 participates in protein folding within the endoplasmic reticulum and is responsible for the processing of secretory proteins. It localizes and functions within the endoplasmic reticulum, one of its roles being mediation of protein folding. ERP29 was also found in extracellular vesicles [37]. It has been described as a tumor suppressor in some cancers, but there are also indications of ERP-29 promoting metastasis [42]. Its role in ovarian cancer has been only sparsely investigated, but its reduced expression has been demonstrated in high-grade serous ovarian cancer compared with benign samples [43].

MAP1A is involved in microtubule organization, being responsible for filamentous cross-bridging between microtubules and other skeletal elements [44]. In ovarian cancer, there are indications that it is a marker of an unfavorable prognosis [45,46].

Although the biological roles of these proteins in ovarian cancer remain incompletely understood, their identification on platelet-derived EVs supports their potential relevance for disease detection and deserves further investigation.

Unexpectedly, we observed no significant differences in the overall levels of CD41+ EVs or CD62P+ platelet-derived EVs between groups. CD62P, or P-selectin, is a cell adhesion protein, predominant in endothelial cells and platelets. While located in alpha-granules, upon platelet activation, it gets exposed to the platelet membrane, mediating coagulation processes, but also interaction with cancer cells [47]. Given previous reports of increased platelet activation in ovarian cancer, we anticipated elevated levels of P-selectin-positive EVs [48,49].

Instead, we observed a differential expression of CD62P+anti-GELS. This finding may indicate selective alterations in platelet activation pathways rather than a generalized increase in platelet activation and highlights the complexity of platelet–tumor interactions, warranting further investigation.

We observed elevated levels of PHB, or prohibitin, in patients with endometriosis compared with other benign lesions. Prohibitin is involved in mitochondrial function and cellular metabolism [50,51]. The first indication of the role of prohibitin in endometriosis was related to a dysfunction of this protein [52]. Later, prohibitin overexpression was also found in granulosa cells in patients with endometriosis [51]. Although the association did not withstand correction for multiple testing, the finding is biologically plausible and may warrant evaluation in larger cohorts.

In feature selection analyses, subjective ultrasound assessment and IOTA classification outperformed both CA125 and platelet-derived EV markers for discrimination between benign and malignant adnexal lesions. This finding reinforces the central role of expert ultrasound evaluation in ovarian cancer diagnostics. Ultrasound diagnosis of borderline tumors and stage I–II ovarian cancer is particularly challenging [53], and our results also confirmed this. Several platelet EV markers remained important contributors to classification models, suggesting that they may provide complementary diagnostic information, particularly in clinically challenging cases. It would be valuable to validate our results in an independent cohort of patients using a different methodological approach, also allowing us to evaluate the transferability to a more common preanalytical platform and pragmatically validate the robustness of the assay. Therefore, independent external validation is needed before clinical application of the proposed biomarkers.

Beyond their potential diagnostic value, the identified platelet-associated proteins may provide insights into the biological interplay between platelets and ovarian cancer. Further investigations of their functional roles may improve our understanding of ovarian cancer progression and potentially reveal novel therapeutic targets.

### Strengths and Limitations

A major strength of this study is its targeted validation of biomarkers previously identified through platelet proteomics. This hypothesis-driven approach is comparable to a probe-based strategy. It enabled evaluation of biologically relevant candidate markers on platelet-derived EVs and provided independent confirmation of their potential diagnostic value for ovarian cancer. Another strength is the use of a clinically well-characterized cohort with histopathologically verified diagnoses. Several limitations should, nevertheless, be acknowledged. These are the cross-sectional design and relatively small sample size of natural reasons, related to disease and stage incidence, and a design with a consecutive inclusion of patients during the study period. This study focused on large platelet-derived EVs in order to validate our previous findings on platelet markers of ovarian cancer. Large EVs are defined by an approximately 0.2–1.0 µm bead-equivalent gate; small EVs (<200 nm) were not analyzed since they were not the focus of this study. Reliable phenotyping of individual small EVs by flow cytometry remains technically challenging, as their scatter and fluorescence signals approach the instrument background and are prone to coincidence (swarm) detection, which would reduce the robustness of marker quantification in a clinical cohort. In addition, biomarker expression was assessed as relative rather than absolute levels. This was by design: fractions of defined platelet EV populations are internally normalized and less sensitive to between-run variation than absolute counts, and since flow cytometry detects only EVs above the instrument detection limit, absolute counts would represent an operationally defined, instrument-dependent value rather than the true EV concentration in plasma. Finally, samples were collected over an extended period (2021–2025). To minimize batch-related variation, analyses were performed in three batches, with bridging samples included across batches, though batch was not included as a covariate in the statistical analyses.

## 5. Conclusions

CD62P+anti-GELS on platelet EVs is associated with ovarian cancer, independent of age and CA125 levels. CD62P+anti-GELS together with CD62P+/CD62P− anti-SRC, CD62P+anti-ACTN4, CD62P−anti-ERP29, and CD62P−anti-MAP1A were identified as candidate markers for distinguishing ovarian cancer from benign and borderline adnexal lesions next to ultrasound evaluation and CA125. These promising candidates require further validation.

## Figures and Tables

**Table 1:** Description of tumor and patient characteristics.

Variable	Benign N = 63	Borderline Tumors N = 20	Ovarian Cancer Stage I–II N = 16	Ovarian Cancer Stage III–IV N = 36	*p*-Value
Age (years), median (Q1–Q3) Mean (SD)	54.00 (41.50–69.00) 54.48 (17.36)	68.00 (62.50–73.25) 65.40 (11.49)	56.00 (50.25–64.25) 55.38 (15.39)	66.00 (54.75–73.00) 61.67 (14.72)	^1^ 0.019
CA125, U/mL, median (Q1–Q3) Mean (SD) [9]	20.25 (13.25–52.25) 49.86 (83.43)	82.00 (36.92–135.00) 120.19 (145.60)	121.00 (75.00–176.00) 254.44 (473.98)	243.00 (112.25–611.75) 1040.97 (2748.07)	^1^ <0.001
Subjective ultrasound evaluation: [11]					
	N = 60	N = 19	N = 13	N = 32	
Benign	39 (65.0%)	2 (10.5%)	2 (15.4%)	0 (0.0%)	^2^ <0.001
Borderline	7 (11.7%)	7 (36.8%)	5 (38.5%)	0 (0.0%)	
Uncertain	7 (11.7%)	7 (36.8%)	1 (7.7%)	4 (12.5%)	
Malignant	7 (11.7%)	3 (15.8%)	5 (38.5%)	25 (78.1%)	
Metastasis	0 (0.0%)	0 (0.0%)	0 (0.0%)	0 (0.0%)	
Nothing abnormal	0 (0.0%)	0 (0.0%)	0 (0.0%)	3 (9.4%)	
IOTA classification: [7]					
	N = 61	N = 20	N = 13	N = 34	
Benign	37 (60.7%)	3 (15.0%)	2 (15.4%)	0 (0.0%)	^2^ <0.001
Uncertain	20 (32.8%)	11 (55.0%)	5 (38.5%)	5 (14.7%)	
Malignant	4 (6.6%)	6 (30.0%)	6 (46.2%)	26 (76.5%)	
Nothing abnormal	0 (0.0%)	0 (0.0%)	0 (0.0%)	3 (8.8%)	

Footnotes: m (a,b) represents median (Q1–Q3); x (s) represents mean (SD); n (p%)—frequency (percentage by group); [M]—number of missings; ^1^—Kruskal–Wallis test; ^2^—Pearson’s χ2 test.

**Table 2:** Median and mean biomarker values multiplied by 1000 for each group.

Markers on Platelet EVs	Benign N = 63	Borderline Tumors N = 20	Ovarian Cancer Stage I–II N = 16	Ovarian Cancer Stage III–IV N = 36	^1^ *p*-Value	^2^ Bonf
CD41+	0.51 (0.20–0.83) 0.55 (0.42)	0.55 (0.24–0.81) 0.62 (0.44)	0.42 (0.16–0.84) 0.81 (0.98)	0.68 (0.31–0.95) 0.81 (0.80)	0.454	1.000
CD41+CD62P+	0.24 (0.11–0.42) 0.29 (0.23)	0.30 (0.12–0.44) 0.32 (0.26)	0.30 (0.08–0.66) 0.48 (0.56)	0.41 (0.25–0.51) 0.44 (0.46)	0.307	1.000
CD41+CD62P−	0.14 (0.06–0.43) 0.28 (0.29)	0.24 (0.07–0.43) 0.30 (0.26)	0.05 (0.01–0.46) 0.35 (0.66)	0.37 (0.02–0.50) 0.39 (0.45)	0.475	1.000
CD62P+anti-RELN	0.05 (0.03–0.11) 0.08 (0.06)	0.07 (0.03–0.10) 0.07 (0.06)	0.07 (0.03–0.14) 0.10 (0.08)	0.07 (0.04–0.09) 0.10 (0.16)	0.916	1.000
CD62P+anti-MAP1A	0.04 (0.01–0.07) 0.05 (0.05)	0.05 (0.03–0.07) 0.05 (0.03)	0.05 (0.01–0.09) 0.07 (0.08)	0.05 (0.02–0.08) 0.07 (0.11)	0.901	1.000
CD62P+anti-MYCT	0.04 (0.02–0.05) 0.04 (0.03)	0.04 (0.03–0.05) 0.05 (0.03)	0.05 (0.02–0.08) 0.07 (0.06)	0.05 (0.03–0.06) 0.06 (0.05)	0.335	1.000
CD62P+anti-ACTN4	0.02 (0.01–0.03) 0.02 (0.02)	0.03 (0.02–0.04) 0.04 (0.03)	0.02 (0.01–0.07) 0.05 (0.06)	0.03 (0.02–0.03) 0.03 (0.03)	0.109	1.000
CD62P+anti-CRKL	0.02 (0.01–0.03) 0.02 (0.02)	0.02 (0.01–0.03) 0.02 (0.02)	0.02 (0.01–0.03) 0.04 (0.06)	0.03 (0.01–0.04) 0.03 (0.04)	0.589	1.000
CD62P+anti-ERP29	0.09 (0.05–0.13) 0.10 (0.08)	0.12 (0.05–0.15) 0.11 (0.08)	0.07 (0.03–0.14) 0.16 (0.24)	0.13 (0.07–0.16) 0.15 (0.12)	0.110	1.000
CD62P+anti-GELS	0.05 (0.02–0.08) 0.06 (0.05)	0.05 (0.03–0.08) 0.07 (0.05)	0.06 (0.01–0.12) 0.09 (0.09)	0.09 (0.05–0.11) 0.12 (0.15)	0.005	0.105
CD62P+anti-PHB	0.10 (0.03–0.17) 0.11 (0.09)	0.08 (0.04–0.15) 0.11 (0.08)	0.10 (0.04–0.17) 0.18 (0.31)	0.14 (0.05–0.16) 0.17 (0.25)	0.751	1.000
CD62P+anti-SRC	0.08 (0.03–0.13) 0.10 (0.09)	0.13 (0.09–0.15) 0.14 (0.10)	0.10 (0.03–0.16) 0.21 (0.33)	0.14 (0.09–0.20) 0.20 (0.29)	0.026	0.546
CD62P− anti-RELN	0.03 (0.01–0.06) 0.05 (0.05)	0.03 (0.01–0.05) 0.03 (0.02)	0.02 (0.00–0.06) 0.07 (0.16)	0.05 (0.03–0.08) 0.07 (0.08)	*0.074*	1.000
CD62P− anti-MAP1A	0.03 (0.01–0.05) 0.04 (0.04)	0.01 (0.01–0.04) 0.03 (0.03)	0.01 (0.00–0.05) 0.03 (0.03)	0.05 (0.01–0.07) 0.07 (0.11)	*0.074*	1.000
CD62P− anti-MYCT	0.04 (0.01–0.08) 0.06 (0.05)	0.06 (0.01–0.11) 0.07 (0.07)	0.02 (0.00–0.07) 0.08 (0.19)	0.09 (0.04–0.15) 0.12 (0.15)	0.009	0.189
CD62P− anti-ACTN4	0.02 (0.01–0.03) 0.02 (0.02)	0.02 (0.01–0.03) 0.02 (0.02)	0.01 (0.00–0.03) 0.03 (0.06)	0.03 (0.02–0.05) 0.04 (0.04)	0.050	1.000
CD62P− anti-CRKL	0.01 (0.00–0.03) 0.02 (0.02)	0.01 (0.01–0.03) 0.02 (0.02)	0.01 (0.00–0.04) 0.05 (0.09)	0.03 (0.01–0.04) 0.02 (0.02)	0.492	1.000
CD62P− anti-ERP29	0.06 (0.04–0.10) 0.08 (0.06)	0.06 (0.03–0.09) 0.07 (0.05)	0.03 (0.01–0.13) 0.17 (0.32)	0.11 (0.05–0.16) 0.14 (0.15)	*0.052*	1.000
CD62P− anti-GELS	0.04 (0.01–0.07) 0.05 (0.05)	0.05 (0.02–0.07) 0.05 (0.04)	0.02 (0.00–0.06) 0.07 (0.13)	0.05 (0.03–0.08) 0.08 (0.13)	0.228	1.000
CD62P− anti-PHB	0.08 (0.02–0.11) 0.10 (0.09)	0.07 (0.02–0.12) 0.09 (0.09)	0.02 (0.01–0.15) 0.21 (0.42)	0.11 (0.05–0.19) 0.15 (0.22)	0.222	1.000
CD62P− anti-SRC	0.05 (0.02–0.10) 0.07 (0.06)	0.06 (0.01–0.09) 0.07 (0.07)	0.03 (0.01–0.10) 0.15 (0.34)	0.09 (0.04–0.20) 0.12 (0.11)	0.127	1.000

Footnotes: m (a,b) represents median (Q1–Q3), x (s) represents mean (SD), ^1^—Kruskal–Wallis test, and ^2^—*p*-values corrected for multiple testing of 21 biomarkers using the Bonferroni correction.

**Table 3:** Odds ratio (OR) of being in the more severe clinical category of the patient group.

Markers on Platelet EVs	Model 0:				Model 1			
Log2	OR (95% CI)	C-Stat	*p*-Value	Bonf	OR (95% CI)	C-Stat	*p*-Value	Bonf
CD41+ (log2)	1.17 (0.94–1.44)	0.557	0.1523	1.0000	1.15 (0.91–1.46)	0.8076	0.2355	1.0000
CD41+ CD62P+ (log2)	1.01 (0.91–1.11)	0.5727	0.9129	1.0000	0.95 (0.85–1.05)	0.8031	0.3309	1.0000
CD41+ CD62P− (log2)	0.96 (0.86–1.07)	0.4887	0.5012	1.0000	1.03 (0.91–1.17)	0.8023	0.6342	1.0000
CD62P+ anti-RELN (log2)	1.06 (0.86–1.31)	0.5178	0.5723	1.0000	1.07 (0.85–1.35)	0.8035	0.5768	1.0000
CD62P+ anti-MAP1A (log2)	1.06 (0.88–1.27)	0.5291	0.5329	1.0000	0.99 (0.81–1.21)	0.8023	0.9033	1.0000
CD62P+ anti-MYCT (log2)	1.23 (0.96–1.58)	0.5663	0.1019	1.0000	1.13 (0.85–1.49)	0.8052	0.3999	1.0000
**CD62P+ anti-ACTN4 (log2)**	**1.33 (1.04–1.69)**	**0.5874**	**0.0226**	0.4745	1.31 (1.00–1.72)	0.8057	0.0523	1.0000
CD62P+ anti-CRKL (log2)	1.13 (0.91–1.40)	0.5463	0.2652	1.0000	1.17 (0.92–1.49)	0.8045	0.2021	1.0000
CD62P+ anti-ERP29 (log2)	1.19 (0.98–1.45)	0.5863	*0.0804*	1.0000	1.14 (0.92–1.42)	0.8027	0.2351	1.0000
**CD62P+ anti-GELS (log2)**	**1.47 (1.16–1.85)**	**0.633**	**0.0013**	**0.0274**	**1.59 (1.21–2.08)**	**0.8236**	**0.0007**	**0.0156**
CD62P+ anti-PHB (log2)	1.15 (0.96–1.36)	0.538	0.1302	1.0000	1.09 (0.89–1.35)	0.8032	0.3963	1.0000
**CD62P+ anti-SRC (log2)**	**1.21 (1.01–1.47)**	**0.6144**	**0.0435**	0.9135	1.17 (0.96–1.43)	0.8055	0.1234	1.0000
CD62P− anti-RELN (log2)	1.02 (0.89–1.17)	0.5352	0.8098	1.0000	1.03 (0.88–1.19)	0.803	0.7390	1.0000
CD62P− anti-MAP1A (log2)	0.99 (0.86–1.13)	0.4852	0.8343	1.0000	1.08 (0.93–1.26)	0.8025	0.3170	1.0000
CD62P− anti-MYCT (log2)	1.07 (0.94–1.21)	0.5815	0.3203	1.0000	1.08 (0.94–1.24)	0.8055	0.2749	1.0000
CD62P− anti-ACTN4 (log2)	1.05 (0.90–1.21)	0.5649	0.5533	1.0000	1.06 (0.91–1.24)	0.8042	0.4595	1.0000
CD62P− anti-CRKL (log2)	1.03 (0.91–1.16)	0.547	0.6638	1.0000	1.03 (0.90–1.18)	0.8024	0.6541	1.0000
CD62P− anti-ERP29 (log2)	1.02 (0.89–1.18)	0.5625	0.7799	1.0000	1.02 (0.88–1.19)	0.802	0.7690	1.0000
CD62P− anti-GELS (log2)	1.01 (0.88–1.15)	0.5255	0.9295	1.0000	1.01 (0.87–1.17)	0.8024	0.9434	1.0000
CD62P− anti-PHB (log2)	1.01 (0.90–1.14)	0.5433	0.8638	1.0000	1.03 (0.91–1.17)	0.8035	0.6348	1.0000
CD62P− anti-SRC (log2)	1.01 (0.88–1.16)	0.5448	0.8735	1.0000	0.99 (0.86–1.16)	0.8023	0.9484	1.0000

Footnotes: Bonferroni correction for 21 markers. Model 1 is adjusted for age and CA125.

**Table 4:** Levels of markers in endometriosis (**A**) and other abdominal malignancy (**B**).

Markers on Platelet EVs	(A)				(B)			
	No Endometriosis	Endometriosis	^1^ *p*-Value	^2^ Bonf	Other Abdominal Cancer	Stage III–IV Ovarian Cancer	^1^ *p*-Value	^2^ Bonf.
CD41+	0.50 (0.22–0.85) 0.56 (0.44)	0.56 (0.17–0.80) 0.52 (0.37)	0.809	1.000	0.56 (0.21–0.90) 0.61 (0.47)	0.68 (0.31–0.95) 0.81 (0.80)	0.466	1.0000
CD41+ CD62P+	0.22 (0.10–0.42) 0.29 (0.24)	0.30 (0.13–0.42) 0.29 (0.19)	0.716	1.000	0.23 (0.11–0.38) 0.29 (0.24)	0.41 (0.25–0.51) 0.44 (0.46)	0.166	1.0000
CD41+ CD62P−	0.14 (0.07–0.45) 0.29 (0.31)	0.13 (0.04–0.39) 0.25 (0.26)	0.597	1.000	0.19 (0.07–0.53) 0.33 (0.34)	0.37 (0.02–0.50) 0.39 (0.45)	0.951	1.0000
CD62P+ anti-RELN	0.05 (0.03–0.11) 0.08 (0.06)	0.05 (0.03–0.09) 0.07 (0.06)	0.821	1.000	0.07 (0.04–0.08) 0.06 (0.04)	0.07 (0.04–0.09) 0.10 (0.16)	0.617	1.0000
CD62P+ anti-MAP1A	0.04 (0.02–0.07) 0.05 (0.05)	0.05 (0.01–0.07) 0.05 (0.05)	0.831	1.000	0.04 (0.02–0.06) 0.05 (0.04)	0.05 (0.02–0.08) 0.07 (0.11)	0.329	1.0000
CD62P+ anti-MYCT	0.04 (0.02–0.06) 0.04 (0.03)	0.05 (0.03–0.05) 0.04 (0.03)	0.557	1.000	0.05 (0.03–0.06) 0.05 (0.02)	0.05 (0.03–0.06) 0.06 (0.05)	0.840	1.0000
CD62P+ anti-ACTN4	0.02 (0.01–0.03) 0.02 (0.02)	0.02 (0.01–0.03) 0.02 (0.02)	0.857	1.000	0.02 (0.01–0.04) 0.03 (0.02)	0.03 (0.02–0.03) 0.03 (0.03)	0.329	1.0000
CD62P+ anti-CRKL	0.02 (0.01–0.03) 0.02 (0.02)	0.03 (0.02–0.03) 0.03 (0.02)	0.262	1.000	0.01 (0.01–0.02) 0.02 (0.02)	0.03 (0.01–0.04) 0.03 (0.04)	0.050	1.0000
CD62P+ anti-ERP29	0.09 (0.04–0.13) 0.11 (0.09)	0.09 (0.05–0.12) 0.09 (0.05)	0.881	1.000	0.03 (0.02–0.07) 0.05 (0.04)	0.13 (0.07–0.16) 0.15 (0.12)	**<0.001**	**<0.001**
CD62P+ anti-GELS	0.05 (0.02–0.08) 0.06 (0.06)	0.05 (0.02–0.08) 0.05 (0.03)	0.600	1.000	0.07 (0.05–0.10) 0.08 (0.04)	0.09 (0.05–0.11) 0.12 (0.15)	0.384	1.0000
**CD62P+ anti-PHB**	0.07 (0.02–0.16) 0.10 (0.09)	0.15 (0.08–0.19) 0.16 (0.10)	**0.047**	0.987	0.07 (0.05–0.15) 0.11 (0.09)	0.14 (0.05–0.16) 0.17 (0.25)	0.292	1.0000
CD62P+ anti-SRC	0.08 (0.04–0.13) 0.10 (0.08)	0.11 (0.03–0.13) 0.11 (0.10)	0.837	1.000	0.10 (0.06–0.17) 0.13 (0.11)	0.14 (0.09–0.20) 0.20 (0.29)	0.242	1.0000
CD62P− anti-RELN	0.03 (0.01–0.06) 0.05 (0.05)	0.05 (0.03–0.07) 0.05 (0.04)	0.343	1.000	0.04 (0.02–0.08) 0.05 (0.04)	0.05 (0.03–0.08) 0.07 (0.08)	0.853	1.0000
CD62P− anti-MAP1A	0.03 (0.01–0.06) 0.04 (0.04)	0.04 (0.01–0.05) 0.04 (0.03)	0.907	1.000	0.02 (0.01–0.03) 0.04 (0.05)	0.05 (0.01–0.07) 0.07 (0.11)	0.091	1.0000
CD62P− anti-MYCT	0.03 (0.01–0.07) 0.05 (0.05)	0.07 (0.02–0.09) 0.07 (0.05)	0.327	1.000	0.06 (0.02–0.11) 0.06 (0.05)	0.09 (0.04–0.15) 0.12 (0.15)	0.091	1.0000
CD62P− anti-ACTN4	0.02 (0.01–0.03) 0.02 (0.02)	0.02 (0.01–0.03) 0.02 (0.01)	0.656	1.000	0.01 (0.00–0.02) 0.02 (0.02)	0.03 (0.02–0.05) 0.04 (0.04)	**0.007**	0.1470
CD62P− anti-CRKL	0.01 (0.00–0.03) 0.02 (0.02)	0.02 (0.00–0.03) 0.02 (0.01)	0.782	1.000	0.03 (0.01–0.04) 0.03 (0.02)	0.03 (0.01–0.04) 0.02 (0.02)	0.605	1.0000
CD62P− anti-ERP29	0.06 (0.04–0.10) 0.07 (0.05)	0.08 (0.03–0.11) 0.09 (0.08)	0.624	1.000	0.06 (0.03–0.13) 0.10 (0.09)	0.11 (0.05–0.16) 0.14 (0.15)	0.279	1.0000
CD62P− anti-GELS	0.04 (0.01–0.07) 0.05 (0.04)	0.06 (0.01–0.07) 0.06 (0.06)	0.611	1.000	0.04 (0.02–0.08) 0.05 (0.05)	0.05 (0.03–0.08) 0.08 (0.13)	0.545	1.0000
CD62P− anti-PHB	0.08 (0.02–0.12) 0.09 (0.09)	0.10 (0.04–0.11) 0.10 (0.10)	0.653	1.000	0.11 (0.02–0.17) 0.13 (0.12)	0.11 (0.05–0.19) 0.15 (0.22)	0.922	1.0000
CD62P− anti-SRC	0.05 (0.02–0.10) 0.07 (0.06)	0.07 (0.02–0.10) 0.07 (0.05)	0.833	1.000	0.07 (0.03–0.13) 0.09 (0.08)	0.09 (0.04–0.20) 0.12 (0.11)	0.381	1.0000

Footnotes: Median and mean biomarker values multiplied by 1000 for each group; m (a,b) represents median (Q1–Q3), and x (s) represents mean (SD); tests used: ^1^—Mann–Whitney U test; ^2^—*p*-values were corrected for multiple testing of 21 biomarkers using Bonferroni correction.

## Data Availability

The data presented in this study are available on request from the corresponding author in an anonymized format due to ethical reasons in relation to the individual patients.

## Supplementary Materials

The following supporting information can be downloaded at https://www.mdpi.com/article/10.3390/cancers18213478/s1: Figure S1. Classification of biomarkers in Boruta algorithm and their level of importance for discrimination between patient groups of benign adnexal lesions, borderline tumors and ovarian cancer stages I–II, including (A) platelet markers on EVs; (B) platelet markers on EVs, age and CA125; (C) platelet markers on EVs, age, CA125 and subjective ultrasound evaluation; and (D) platelet markers, age, CA125 and IOTA ultrasound. Table S1. Description of the studied proteins, antibodies and antibody cocktails. (A) Description of antibodies and dilutions. (B) Description of the three antibody cocktails with conjugates. (C) Characteristics of studied proteins. Table S2. Odds ratios (ORs) of being in the more severe clinical category of the patient groups. Table S3. Odds ratios (ORs) of being endometriosis compared with other benign lesions. Table S4. Odds ratios (ORs) of being advanced ovarian cancer stage III–IV compared with other abdominal malignancies.
