# Supplementary material for: Platelet-Derived Extracellular Vesicles for the Diagnosis of Ovarian Cancer
Source: Cancers (Basel). 2026 Oct 28;18(21):3478. doi: 10.3390/cancers18213478 (PMC13762264; doi:10.3390/cancers18213478)
Supplement: Supplementary file 1 [file cancers-18-03478-s001.zip › cancers-4624736-supplementary.pdf]

Platelet-Derived Extracellular Vesicles for the Diagnosis of Ovarian Cancer

Evelina Karlsson [1], Katja Gabrysch [2], Karin Stålberg [1], Inger Sundström-Poromaa [1], Fariborz Mobarrez [3,†] and Marta Lomnytska [1,*,†]

Table S1. Description of the studied proteins, antibodies and antibody cocktails.

(A) Description of antibodies and dilutions.

Target	Clone	Host and Reactivity	Conjugate (in-House Labeling Kit)	Supplier	Cat. No.	Conc. Used
1	2	3	4	5	6	7
CD41	P2	Mouse anti-human (IgG1)	APC (factory-conjugated)	Beckman Coulter	B16894	5 µL/test
CD62P	CLBThromb/6	Mouse anti-human (IgG1)	PE (factory-conjugated)	Beckman Coulter	IM1759U	5 µL/test
RELN	G10	Mouse anti-human/mouse/rat (IgG1)	Alexa Fluor 488 (Lightning-Link, ab236553)	Abcam	ab78540	8 µg/mL
MAP1A	EPR18993	Rabbit anti-human/mouse/rat (rec. mAb)	PE/Cy5.5 (Lightning-Link, ab102899)	Abcam	ab184349	8 µg/mL
MYCT1	Polyclonal	Rabbit anti-human/mouse/rat	Alexa Fluor 700 (Lightning-Link, ab269824)	Abcam	ab139945	8 µg/mL
ACTN4	Polyclonal	Rabbit anti-human	Alexa Fluor 488 (Lightning-Link, ab236553)	Abcam	ab59468	8 µg/mL
CRKL	Y244	Rabbit anti-human/mouse/rat (rec. mAb)	PE/Cy5.5 (Lightning-Link, ab102899)	Abcam	ab32018	8 µg/mL
ERP29	Polyclonal	Rabbit anti-human (+ other sp.)	Alexa Fluor 700 (Lightning-Link, ab269824)	Abcam	ab11420	8 µg/mL
GELS (GSN)	GS-2C4	Mouse anti-human (IgG1)	Alexa Fluor 488 (Lightning-Link, ab236553)	Abcam	ab11081	8 µg/mL
PHB	PHB/1881	Mouse anti-human	PE/Cy5.5 (Lightning-Link, ab102899)	Abcam	ab224653	8 µg/mL
SRC	EPR5496	Rabbit anti-human/mouse/rat (rec. mAb)	Alexa Fluor 700 (Lightning-Link, ab269824)	Abcam	ab109381	8 µg/mL
CD41	P2	Mouse anti-human (IgG1)	APC (factory-conjugated)	Beckman Coulter	B16894	5 µL/test

CD62P	CLBThromb/6	Mouse anti-human (IgG1)	PE (factory-conjugated)	Beckman Coulter	IM1759U	5 µL/test
RELN	G10	Mouse anti-human/mouse/rat (IgG1)	Alexa Fluor 488 (Lightning-Link, ab236553)	Abcam	ab78540	8 µg/mL
MAP1A	EPR18993	Rabbit anti-human/mouse/rat (rec. mAb)	PE/Cy5.5 (Lightning-Link, ab102899)	Abcam	ab184349	8 µg/mL
MYCT1	Polyclonal	Rabbit anti-human/mouse/rat	Alexa Fluor 700 (Lightning-Link, ab269824)	Abcam	ab139945	8 µg/mL
ACTN4	Polyclonal	Rabbit anti-human	Alexa Fluor 488 (Lightning-Link, ab236553)	Abcam	ab59468	8 µg/mL
CRKL	Y244	Rabbit anti-human/mouse/rat (rec. mAb)	PE/Cy5.5 (Lightning-Link, ab102899)	Abcam	ab32018	8 µg/mL
ERP29	Polyclonal	Rabbit anti-human (+ other sp.)	Alexa Fluor 700 (Lightning-Link, ab269824)	Abcam	ab11420	8 µg/mL
GELS (GSN)	GS-2C4	Mouse anti-human (IgG1)	Alexa Fluor 488 (Lightning-Link, ab236553)	Abcam	ab11081	8 µg/mL
PHB	PHB/1881	Mouse anti-human	PE/Cy5.5 (Lightning-Link, ab102899)	Abcam	ab224653	8 µg/mL
SRC	EPR5496	Rabbit anti-human/mouse/rat (rec. mAb)	Alexa Fluor 700 (Lightning-Link, ab269824)	Abcam	ab109381	8 µg/mL

Footnotes:

Conjugates: APC = allophycocyanin; PE = phycoerythrin; AF = Alexa Fluor; PE/Cy5.5 = phycoerythrin–cyanine5.5 tandem.

CD41 and CD62P were obtained as factory-conjugated reagents (Beckman Coulter, ASR class). The nine anti-protein antibodies (Abcam) were conjugated in-house using Lightning-Link® kits: Alexa Fluor® 488 (ab236553), PE/Cy5.5® (ab102899) and Alexa Fluor® 700 (ab269824). Concentration refers to the working concentration of each conjugate used in the staining reaction.

(B) Description of the three antibody cocktails with conjugates.

Antibody Cocktail 1	Antibody Cocktail 2	Antibody Cocktail 3
CD41 APC	CD41 APC	CD41 APC
CD62 PE	CD62 PE	CD62 PE

Anti-RELN AF 488	Anti-ACTN4 AF 488	Anti-GELS AF 488
Anti-MAP1A PE-CY5.5	Anti-CRKL PE-CY5.5	Anti-PHB PE-CY5.5
Anti-MYCT AF 700	Anti-ERP29 AF 700	Anti-SRC AF 700

Footnotes: Conjugates: APC=allophycocyanin, PE=phycoerythrin, AF=Alexa Fluor, and PE-CY=phycoerythrin–cyanin.

(C) Characteristics of studied proteins.

UniProt ID	Gene Ontology Name	Protein Name
Q96BG6	ACTN4	Alpha actinin 4
P46109	CRKL	Crk-like protein
P30040	ERP29	Endoplasmic reticulum resident protein 29
P06396	GELS	Gelsolin
P78559	MAP1A	Microtubule-associated protein 1A
Q8N699	MYCT1	Myc target protein 1
P35232	PHB	Prohibitin
P78509	RELN	Reelin
P12931	SRC	Protooncogene tyrosine-protein kinase Src

Figure S1. Classification of biomarkers in Boruta algorithm and their level of importance for discrimination between patient groups of benign adnexal lesions, borderline tumors and ovarian cancer stages I–II, including (A) platelet markers on EVs; (B) platelet markers on EVs, age and CA125; (C) platelet markers on EVs, age, CA125 and subjective ultrasound evaluation; and (D) platelet markers, age, CA125 and IOTA ultrasound.

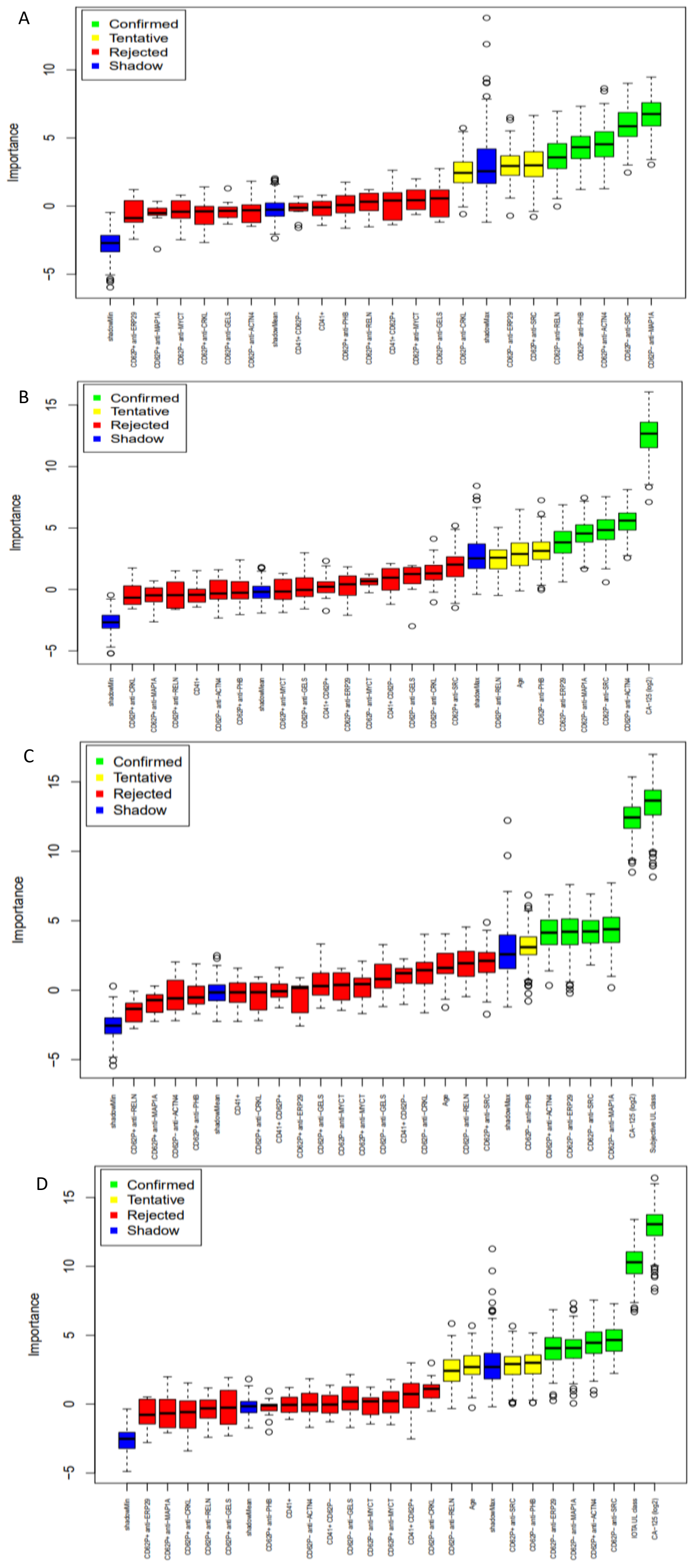

Table S2. Odds ratios (ORs) of being in the more severe clinical category of the patient groups.

Markers on Platelet EVs	Model 0				Model 1				Model 2				
	OR(95% CI)	C-Stat	p-Value	Bonf	OR(95% CI)	C-Stat	p-Value	Bonf	OR(95% CI)	C-Stat	p-Value	Bonf	
CD41+ (log2)	1.18(0.95--1.46)	0.5608	0.1355	1.0000	0.95(0.73--1.24)	0.8799	0.7096	1.0000	0.92(0.70--1.21)	0.8726	0.5501	1.0000	
CD41+ CD62P+ (log2)	1.00(0.91--1.11)	0.5825	0.9181	1.0000	0.91(0.82--1.02)	0.8803	0.1178	1.0000	0.89(0.79--0.99)	0.877	0.0359	0.7536	
CD41+ CD62P- (log2)	0.95(0.85--1.07)	0.4928	0.4092	1.0000	1.01(0.88--1.17)	0.8799	0.8494	1.0000	0.98(0.85--1.13)	0.8727	0.8049	1.0000	
CD62P+ anti-RELN (log2)	1.14(0.91--1.41)	0.5321	0.2528	1.0000	1.05(0.80--1.37)	0.88	0.7274	1.0000	1.06(0.82--1.38)	0.873	0.6601	1.0000	
CD62P+ anti-MAP1A (log2)	1.08(0.90--1.30)	0.5404	0.4122	1.0000	0.84(0.67--1.05)	0.8832	0.1219	1.0000	0.84(0.67--1.04)	0.8749	0.1110	1.0000	
CD62P+ anti-MYCT (log2)	1.25(0.97--1.61)	0.573	0.0865	1.0000	0.89(0.64--1.22)	0.8787	0.4560	1.0000	0.85(0.61--1.17)	0.8707	0.3147	1.0000	
**CD62P+ anti-ACTN4 (log2)**	**1.38(1.08--1.77)**	**0.6023**	**0.0113**	0.2370	0.99(0.72--1.35)	0.8799	0.9330	1.0000	1.07(0.79--1.47)	0.8718	0.6548	1.0000	
CD62P+ anti-CRKL (log2)	1.12(0.90--1.39)	0.5399	0.3167	1.0000	0.96(0.74--1.24)	0.8797	0.7286	1.0000	0.91(0.70--1.20)	0.8718	0.5131	1.0000	
CD62P+ anti-ERP29 (log2)	1.19(0.97--1.45)	0.5845	0.0922	1.0000	1.10(0.87--1.38)	0.8805	0.4170	1.0000	1.07(0.86--1.34)	0.871	0.5348	1.0000	
**CD62P+ anti-GELS (log2)**	**1.47(1.16--1.86)**	**0.6309**	**0.0015**	**0.0322**	**1.35(1.01--1.82)**	**0.8821**	**0.0439**	0.9223	***1.31(0.97--1.77)***	***0.8746***	***0.0754***	1.0000	
CD62P+ anti-PHB (log2)	1.14(0.96--1.36)	0.5404	0.1415	1.0000	0.98(0.77--1.24)	0.88	0.8705	1.0000	0.94(0.75--1.17)	0.8719	0.5640	1.0000	
CD62P+ anti-SRC (log2)	1.20(0.99--1.46)	0.6106	0.0572	1.0000	0.98(0.79--1.22)	0.8803	0.8709	1.0000	0.96(0.76--1.21)	0.8728	0.7222	1.0000	
CD62P- anti-RELN (log2)	1.01(0.88--1.16)	0.5325	0.8894	1.0000	0.94(0.80--1.12)	0.8802	0.4905	1.0000	0.89(0.75--1.06)	0.8737	0.2027	1.0000	
CD62P- anti-MAP1A (log2)	0.97(0.85--1.11)	0.4943	0.6895	1.0000	0.96(0.80--1.14)	0.8805	0.6176	1.0000	0.91(0.75--1.09)	0.8745	0.2957	1.0000	
CD62P- anti-MYCT (log2)	1.06(0.93--1.20)	0.5745	0.4167	1.0000	0.97(0.83--1.14)	0.8805	0.7489	1.0000	0.92(0.79--1.08)	0.8736	0.3284	1.0000	
CD62P- anti-ACTN4 (log2)	1.03(0.89--1.19)	0.5553	0.7118	1.0000	0.95(0.80--1.14)	0.8804	0.5883	1.0000	0.91(0.76--1.10)	0.8723	0.3414	1.0000	
CD62P- anti-CRKL (log2)	1.03(0.91--1.17)	0.5573	0.6044	1.0000	0.96(0.82--1.11)	0.8801	0.5647	1.0000	0.89(0.75--1.05)	0.8744	0.1634	1.0000	
CD62P- anti-ERP29 (log2)	1.00(0.86--1.15)	0.4544	0.9655	1.0000	0.88(0.74--1.05)	0.8806	0.1506	1.0000	0.79(0.65--0.97)	0.8793	0.0250	0.5246	
CD62P- anti-GELS (log2)	0.99(0.86--1.14)	0.4861	0.8748	1.0000	0.88(0.74--1.06)	0.8808	0.1708	1.0000	0.84(0.69--1.01)	0.8771	0.0705	1.0000	
CD62P- anti-PHB (log2)	0.99(0.88--1.12)	0.4684	0.9236	1.0000	0.91(0.78--1.05)	0.8804	0.2005	1.0000	0.88(0.75--1.03)	0.8757	0.1191	1.0000	
CD62P- anti-SRC (log2)	1.01(0.88--1.17)	0.5459	0.8457	1.0000	0.89(0.75--1.06)	0.8809	0.1942	1.0000	0.87(0.72--1.04)	0.8767	0.1198	1.0000	

Footnotes: Bonferroni correction for 21 markers. Model 1 is adjusted for age, CA125 and subjective ultrasound; Model 2 is adjusted for age, CA125 and ultrasound by IOTA.

Table S3. Odds ratios (ORs) of being endometriosis compared with other benign lesions.

Markers on Platelet EVs	Model 0				Model 1			
Biomarker (Log 2)	OR(95% CI)	C-Stat	p-Value	Bonf	OR(95% CI)	C-Stat	p-Value	Bonf
CD41+	0.88(0.61--1.28)	0.5213	0.4907	1.0000	0.89(0.60--1.32)	0.6822	0.5579	1.0000
CD41+ CD62P+	1.02(0.87--1.27)	0.5312	0.8206	1.0000	1.02(0.86--1.27)	0.6915	0.8436	1.0000
CD41+ CD62P-	0.91(0.72--1.15)	0.5452	0.4034	1.0000	0.94(0.74--1.21)	0.6842	0.6217	1.0000
CD62P+ anti-RELN	0.98(0.65--1.51)	0.5199	0.9384	1.0000	0.99(0.64--1.53)	0.6902	0.9493	1.0000
CD62P+ anti-MAP1A	0.98(0.70--1.41)	0.5186	0.9312	1.0000	0.95(0.66--1.39)	0.6782	0.7903	1.0000
CD62P+ anti-MYCT	1.18(0.76--1.94)	0.5505	0.4804	1.0000	1.14(0.71--1.95)	0.6888	0.5992	1.0000
CD62P+ anti-ACTN4	1.02(0.65--1.66)	0.516	0.9284	1.0000	0.96(0.59--1.59)	0.6862	0.8622	1.0000
CD62P+ anti-CRKL	1.11(0.77--1.73)	0.5957	0.5929	1.0000	1.10(0.73--1.80)	0.6941	0.6739	1.0000
CD62P+ anti-ERP29	1.00(0.70--1.49)	0.4867	0.9951	1.0000	0.86(0.57--1.31)	0.6848	0.4524	1.0000
CD62P+ anti-GELS	0.93(0.65--1.37)	0.5452	0.7094	1.0000	0.89(0.61--1.33)	0.6875	0.5503	1.0000
**CD62P+ anti-PHB**	**1.55(1.08--2.52)**	**0.6676**	**0.0403**	0.8457	1.54(1.04--2.59)	0.7553	0.0575	1.0000
CD62P+ anti-SRC	1.04(0.71--1.55)	0.518	0.8432	1.0000	0.97(0.65--1.46)	0.6902	0.8665	1.0000
CD62P- anti-RELN	1.15(0.81--1.68)	0.5811	0.4452	1.0000	1.19(0.84--1.74)	0.6941	0.3428	1.0000
CD62P- anti-MAP1A	1.01(0.72--1.44)	0.5106	0.9558	1.0000	1.02(0.71--1.47)	0.6888	0.9292	1.0000
CD62P- anti-MYCT	1.12(0.82--1.60)	0.5838	0.4941	1.0000	1.08(0.78--1.55)	0.6822	0.6346	1.0000
CD62P- anti-ACTN4	0.87(0.60--1.26)	0.5386	0.4438	1.0000	0.89(0.60--1.30)	0.6795	0.5353	1.0000
CD62P- anti-CRKL	1.00(0.75--1.35)	0.4761	0.9843	1.0000	0.97(0.72--1.33)	0.6862	0.8635	1.0000
CD62P- anti-ERP29	1.04(0.69--1.61)	0.5419	0.8555	1.0000	1.11(0.73--1.74)	0.6782	0.6274	1.0000
CD62P- anti-GELS	1.02(0.74--1.44)	0.5439	0.8911	1.0000	1.12(0.80--1.62)	0.6809	0.5141	1.0000
CD62P- anti-PHB	1.04(0.76--1.46)	0.5386	0.8016	1.0000	1.11(0.80--1.59)	0.6809	0.5495	1.0000
CD62P- anti-SRC	0.97(0.64--1.48)	0.4814	0.8776	1.0000	0.96(0.63--1.47)	0.6795	0.8592	1.0000

Footnotes: Model 0 includes the biomarker as a continuous variable; Model 1 includes the biomarker and age; and the C-statistic for Model 1 without any biomarker is 0.6928.

Table S4. Odds ratios (ORs) of being advanced ovarian cancer stage III–IV compared with other abdominal malignancies.

Markers on Platelet EVs	Model 0				Model 1			
Biomarker (Log2)	OR(95% CI)	C-Stat	p-Value	Bonf	OR(95% CI)	C-Stat	p-Value	Bonf
CD41+	1.18(0.85--1.67)	0.5614	0.3271	1.0000	1.20(0.86--1.75)	0.7284	0.2928	1.0000
CD41+ CD62P+	1.04(0.93--1.17)	0.6155	0.4435	1.0000	1.03(0.91--1.17)	0.7099	0.5953	1.0000
CD41+ CD62P-	0.92(0.75--1.12)	0.5058	0.4357	1.0000	0.99(0.78--1.22)	0.7145	0.9040	1.0000
CD62P+ anti-RELN	1.10(0.75--1.62)	0.5424	0.6318	1.0000	1.13(0.76--1.71)	0.7114	0.5307	1.0000
CD62P+ anti-MAP1A	1.12(0.78--1.61)	0.5819	0.5302	1.0000	1.06(0.72--1.60)	0.716	0.7571	1.0000
CD62P+ anti-MYCT	0.96(0.59--1.50)	0.5175	0.8543	1.0000	0.88(0.50--1.45)	0.716	0.6168	1.0000
CD62P+ anti-ACTN4	1.46(0.92--2.45)	0.5819	0.1230	1.0000	1.32(0.78--2.34)	0.6975	0.3139	1.0000
CD62P+ anti-CRKL	1.34(0.93--1.99)	0.6623	0.1254	1.0000	1.25(0.86--1.89)	0.7299	0.2519	1.0000
**CD62P+ anti-ERP29**	**2.43(1.53--4.38)**	**0.845**	**0.0008**	**0.0174**	**2.82(1.64--5.81)**	**0.8596**	**0.0010**	**0.0211**
CD62P+ anti-GELS	1.40(0.89--2.35)	0.5731	0.1632	1.0000	1.48(0.92--2.63)	0.7315	0.1339	1.0000
CD62P+ anti-PHB	1.05(0.75--1.47)	0.5877	0.7497	1.0000	1.06(0.70--1.55)	0.7099	0.7695	1.0000
CD62P+ anti-SRC	1.16(0.81--1.69)	0.598	0.4266	1.0000	1.16(0.80--1.73)	0.7145	0.4332	1.0000
CD62P- anti-RELN	1.02(0.75--1.37)	0.5161	0.8690	1.0000	1.10(0.79--1.49)	0.6975	0.5533	1.0000
CD62P- anti-MAP1A	1.16(0.89--1.53)	0.6404	0.2689	1.0000	1.29(0.97--1.74)	0.7577	0.0792	1.0000
CD62P- anti-MYCT	1.15(0.89--1.52)	0.6404	0.2746	1.0000	1.21(0.91--1.61)	0.7191	0.1817	1.0000
CD62P- anti-ACTN4	1.32(1.03--1.75)	0.7208	0.0356	0.7479	1.46(1.12--1.98)	0.8025	0.0086	0.1798
CD62P- anti-CRKL	0.95(0.71--1.22)	0.5439	0.6765	1.0000	0.99(0.73--1.30)	0.716	0.9413	1.0000
CD62P- anti-ERP29	1.06(0.80--1.38)	0.5906	0.6845	1.0000	1.11(0.82--1.50)	0.7145	0.4748	1.0000
CD62P- anti-GELS	1.03(0.78--1.36)	0.5512	0.8098	1.0000	1.06(0.77--1.41)	0.7114	0.7158	1.0000
CD62P- anti-PHB	1.03(0.80--1.31)	0.5088	0.8258	1.0000	1.14(0.87--1.50)	0.7253	0.3173	1.0000
CD62P- anti-SRC	1.03(0.77--1.36)	0.5731	0.8424	1.0000	1.04(0.74--1.42)	0.7037	0.8159	1.0000

Footnotes: Model 0 includes the biomarker as a continuous variable; Model 1 includes the biomarker, age, and CA125 (log2); and the C-statistic for Model 1 without any biomarker is 0.7114.
